# Knee and foot contracture occur earliest in children with cerebral palsy: a longitudinal analysis of 2,693 children

**DOI:** 10.1080/17453674.2020.1848154

**Published:** 2020-11-24

**Authors:** Erika Cloodt, Philippe Wagner, Henrik Lauge-Pedersen, Elisabet Rodby-Bousquet

**Affiliations:** a Department of Clinical Sciences Lund, Orthopaedics, Lund University, Lund;; b Department of Research and Development, Region Kronoberg, Växjö;;; c Centre for Clinical Research Västerås, Uppsala University-Region Västmanland, Västerås, Sweden

## Abstract

Background and purpose — Joint contracture is a common problem among children with cerebral palsy (CP). To prevent severe contracture and its effects on adjacent joints, it is crucial to identify children with a reduced range of motion (ROM) early. We examined whether significant hip, knee, or foot contracture occurs earliest in children with CP.

Patients and methods — This was a longitudinal study involving 27,230 measurements obtained for 2,693 children (59% boys, 41% girls) with CP born 1990 to 2018 and registered before 5 years of age in the Swedish surveillance program for CP. The analysis was based on 4,751 legs followed up for an average of 5.0 years. Separate Kaplan–Meier (KM) curves were drawn for each ROM to illustrate the proportions of contracture-free legs at a given time during the follow-up. Using a clustered bootstrap method and considering the child as the unit of clustering, 95% pointwise confidence intervals were generated for equally spaced time points every 2.5 years for each KM curve.

Results — Contracture developed in 34% of all legs, and the median time to the first contracture was 10 years from the first examination. Contracture was most common in children with a higher Gross Motor Function Classification System (GMFCS) level. The first contracture was a flexion contracture preventing dorsiflexion in children with GMFCS level I or II and preventing knee extension in children with GMFCS level III to V.

Interpretation — Early interventions to prevent knee and foot contractures in children with CP should be considered.

Joint contracture is a common problem in children with cerebral palsy (CP) (Rosenbaum et al. 2007). Spasticity, muscle imbalance, inability to move, and muscle pathology constrain normal muscle growth and lead to dynamic contracture followed by static joint contracture over time because of tight muscles surrounding the joints (Barrett and Lichtwark 2010). Children with CP exhibit increased sarcomere length and reduced number of satellite cells, both of which affect the ability to maintain muscle length during development and bone growth (Barrett and Lichtwark 2010, Smith et al. 2013). The risk of contracture increases with age and higher level on the Gross Motor Function Classification System (GMFCS) (Nordmark et al. 2009). Some 60% of adults with CP experience contracture in the lower limbs (Agustsson et al. 2018).

Joint contracture prevents mechanical alignment of the joints, which affects standing and lying positions, and the quality and energy cost of gait (Raja et al. 2007). Contracture of the foot, knee, or hip joint may also affect adjacent joints and lead to severe postural asymmetries, windswept hips, and scoliosis (Agustsson et al. 2017, 2018, Pettersson et al. 2020). Contracture is often associated with pain, which occurs most frequently in the lower limbs of children with CP (Alriksson-Schmidt and Hägglund 2016, Blackman et al. 2018). To prevent severe joint contracture and reduce its effects on adjacent joints, it is crucial to identify children with reduced range of motion (ROM) to begin targeted treatment early (Chan and Miller 2014).

We analyzed whether contracture preventing hip extension, knee extension, or foot dorsiflexion occurs first in children with CP with GMFCS level I to V.

## Patients and methods

**Figure 1.**
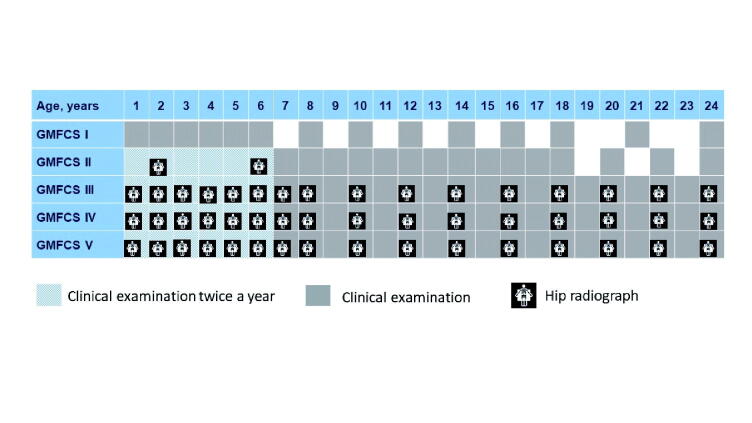
Guidelines for clinical examination and hip radiograph within the Swedish Cerebral Palsy Follow-up Program (CPUP).

This was a prospective study based on register data from the Swedish Cerebral Palsy Follow-up Program (CPUP), which includes > 95% of all children with CP in Sweden. We analyzed all measurements reported from the start of the program in October 1994 until the end of June 2018 and included all children born 1990 to 2018 and registered in the CPUP before 5 years of age. Children registered at 5 years of age or later were excluded. Children included in the CPUP were examined every 6 months, once a year, or every other year, depending on their age and GMFCS level (Figure 1).

The systematic follow-up includes several variables such as reports of surgery, CP subtype, and clinical examinations of passive ROM and gross motor function. The full protocol is available at: https://cpup.se/in-english/manuals-and-evaluation-forms/.

Gross motor function was classified by the child’s physiotherapist into level I to V according to the expanded and revised version of the GMFCS (Palisano et al. 2008). Passive hip extension, knee extension, and dorsiflexion were assessed by goniometric measurements in standardized positions. Hip extension was measured with the child in the prone position with legs over the end of the examining table and the pelvis straight. Knee extension and foot dorsiflexion were measured with the child in the supine position with the hip and knee extended.

Contracture was defined as hip and knee flexion contracture of 10° or more and plantarflexion contracture of at least 10° in extension of the hip, knee, or foot. Children with unilateral spastic CP were identified, and only the affected side was used in the analyses. For children with ataxic, dyskinetic, or spastic bilateral CP, both legs were included.

Each leg that underwent soft tissue surgery or bony surgery and both legs of children with an intrathecal baclofen pump (ITB) or who underwent a selective dorsal rhizotomy (SDR) operation before the onset of the first contracture were censored from the analyses at the date of surgery. If information on which leg was operated on was missing, both legs were censored at the date of surgery.

Reports of spasticity-reducing surgery, such as that to insert an ITB or SDR, and the date of surgery or pump insertion were extracted. Surgery of the lower limbs was recorded in the database and was grouped into either soft tissue surgery or bony surgery for the hip, knee, or foot along with the date of surgery for the left and right leg. Examples of the soft tissue surgeries performed were adductor tenotomy, hamstring or Achilles tendon lengthening, and tendon or muscles transfer. Examples of the bony surgeries reported were osteotomy, physiodesis, or arthrodesis. Surgery on the upper extremity or the spine, fracture, extraction of osteosynthesis material, or treatment with botulinum toxin injection were not censored and data for children who received these treatments were retained in the analyses.

### Statistics

Hip extension, knee extension, and foot dorsiflexion were dichotomized into 2 groups: no contracture and contracture. Both legs for each child were then followed up from inclusion in the CPUP surveillance program until the last examination or the date of surgery. The follow-up time was calculated for each leg for each child, and separate Kaplan–Meier (KM) curves were drawn for each leg’s ROM to illustrate the proportions of contracture-free legs at a given time during the follow-up. For children with unilateral spastic CP, only the affected side was included in the analyses. Using a clustered bootstrap method and considering the child as the unit of clustering, 95% pointwise confidence intervals (CI) were generated for equally spaced time points every 2.5 years for each KM curve. IBM SPSS Statistics (version 25.0; IBM Corp, Armonk, NY, USA), STATA (version 14, Stata-Corp, College Station, TX, USA), and R (R Foundation for Statistical Computing, Vienna, Austria) were used for the statistical analyses. Categorical variables are described by frequency (n) and percentage (%).

### Ethics, funding, and potential conflicts of interest

The study was approved by the Medical Research Ethics Committee in Lund (383/2007, 443-99), and permission was obtained to extract data from the CPUP registry. The study received funding from Stiftelsen för bistånd åt rörelsehindrade i Skåne, Promobilia, Forte and Region Kronoberg. The funding sources had no decision-making role or influence on the study design, data collection, data analysis, data interpretation, or writing of the report. The authors declare that they have no conflicts of interest.

## Results

2,693 children (1,598 boys, 1,095 girls) with an examination before the age of 5 years were reported between 1994 and 2018. The analysis was based on 4,751 legs followed up for an average of 5.0 years (only the affected leg was included for children with unilateral CP). Contractures were in general most common at higher GMFCS levels (Table 1). There were 27,230 measurement occasions.

**Table 1.** Contracture events and number of person-years according to the Gross Motor Function Classification System I–V (GMFCS)

GMFCS level	n (%)	Contracture, legs (%)	Person-years (mean)
I	1,901 (40)	237 (13)	11,450 (6.0)
II	765 (16)	183 (24)	4,495 (5.9)
III	540 (11)	247 (46)	2,250 (4.2)
IV	752 (16)	436 (58)	3,166 (4.2)
V	793 (17)	499 (63)	2,406 (3.0)
Total	4,751 (	1,602 (34)	23,768 (5.0)

**Figure 2.**
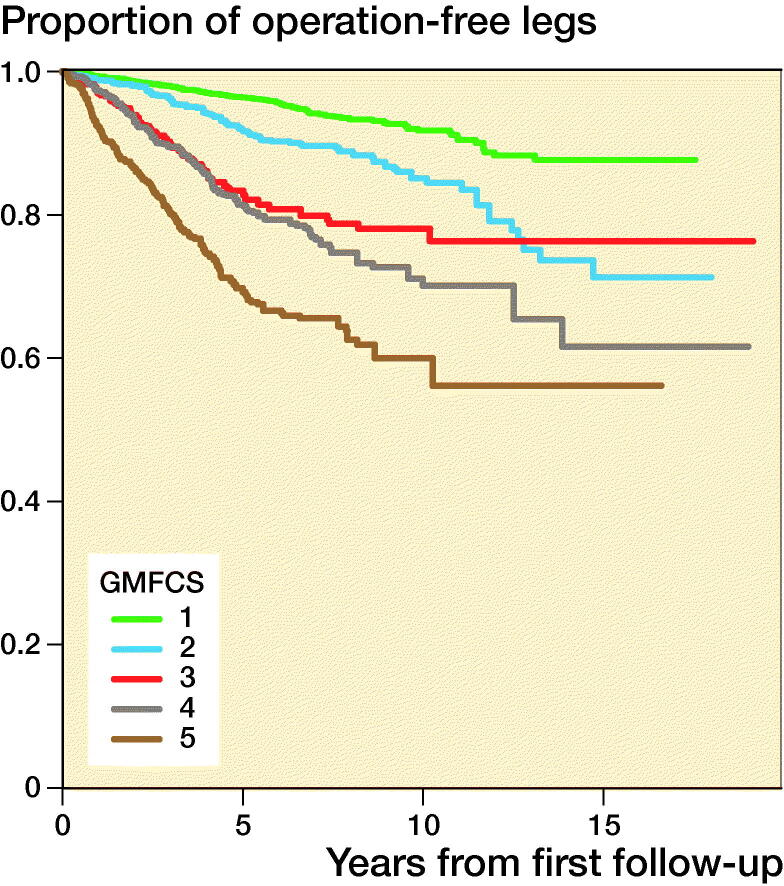
Proportions of operation-free legs from the time of the first measurement for each GMFCS level.

Within the 10 years of follow-up, 937 legs (20%) had been operated on. The most common operation was soft tissue surgery of the hip (316 legs, 6.7%), followed by soft tissue surgery of the foot (201 legs, 4.2%), and bony surgery (osteotomy) of the hip (151 legs 3.2%). The median ages were 4 years for soft tissue surgery of the hip, 7 years for soft tissue surgery of the foot, and 6.5 years for bony surgery of the hip (Table 2). The proportions of legs not operated on in children with different GMFCS levels are presented in Figure 2.

**Table 2.** Age at first hip, knee, and foot operation

		Age at first operation	
Type of surgery	Median	1st quartile	3rd quartile
Hip, soft tissue	4.0	3.0	6.0
Hip, bony	6.5	4.0	10
Knee, soft tissu	9.0	7.0	12
Knee, bony	10	5.5	12
Foot, soft tissu	7.0	5.0	10
Foot, bony	11	8.5	14

1,602 contractures were recorded. A contracture developed in 34% of all legs, and the median time to the first contracture was 10 years from the first examination within the follow-up program. Contracture occurred most frequently in children with a higher GMFCS level. The first contracture to occur in children with GMFCS level I or II was a foot or ankle contracture, and a knee contracture was the first contracture to develop in children with GMFCS level III to V. Proportions of legs free from hip, knee, and foot contracture in each GMFCS level are presented in Table 3 and with separate KM curves in Figure 3.

Figure 3.Proportions of legs free from hip, knee, and foot contracture, stratified by GMFCS level and 95% pointwise confidence intervals for equally spaced time points every 2.5 years during the follow-up.

**Figure:**
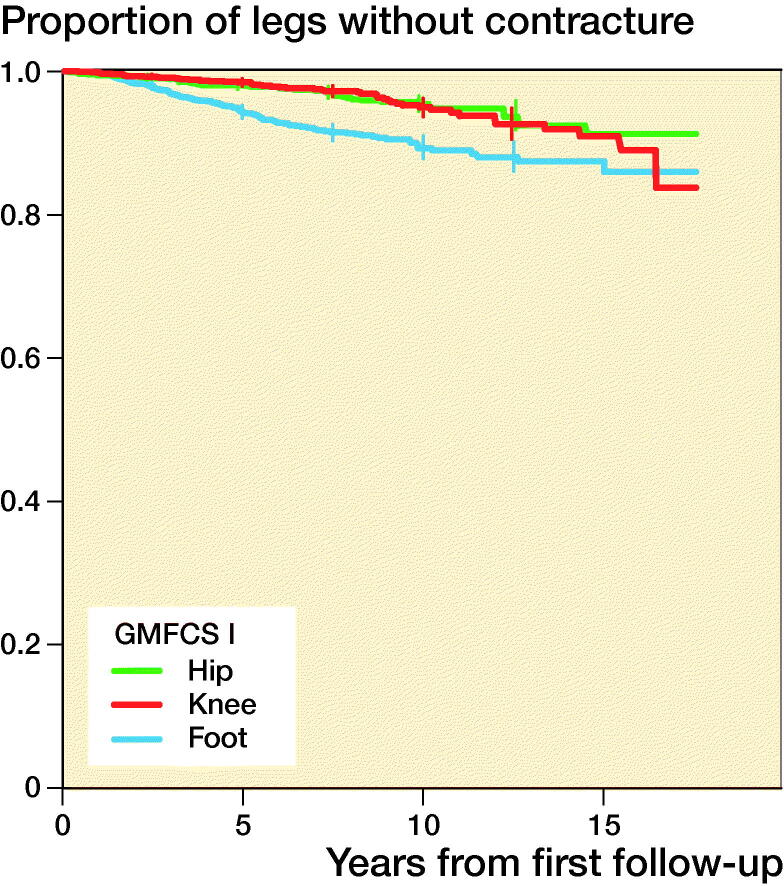

**Figure:**
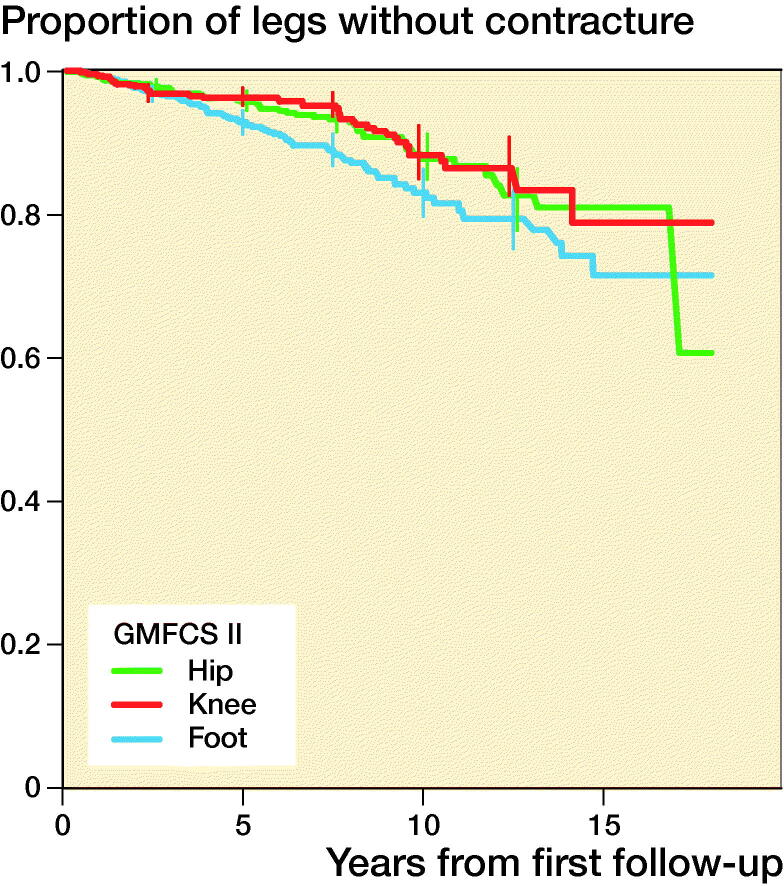

**Figure:**
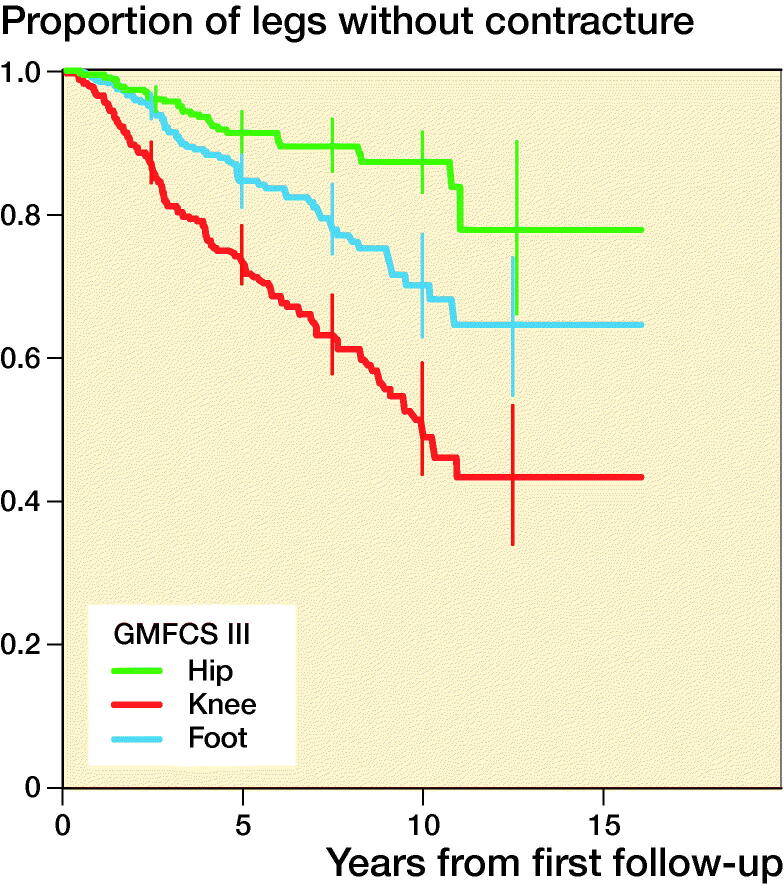

**Figure:**
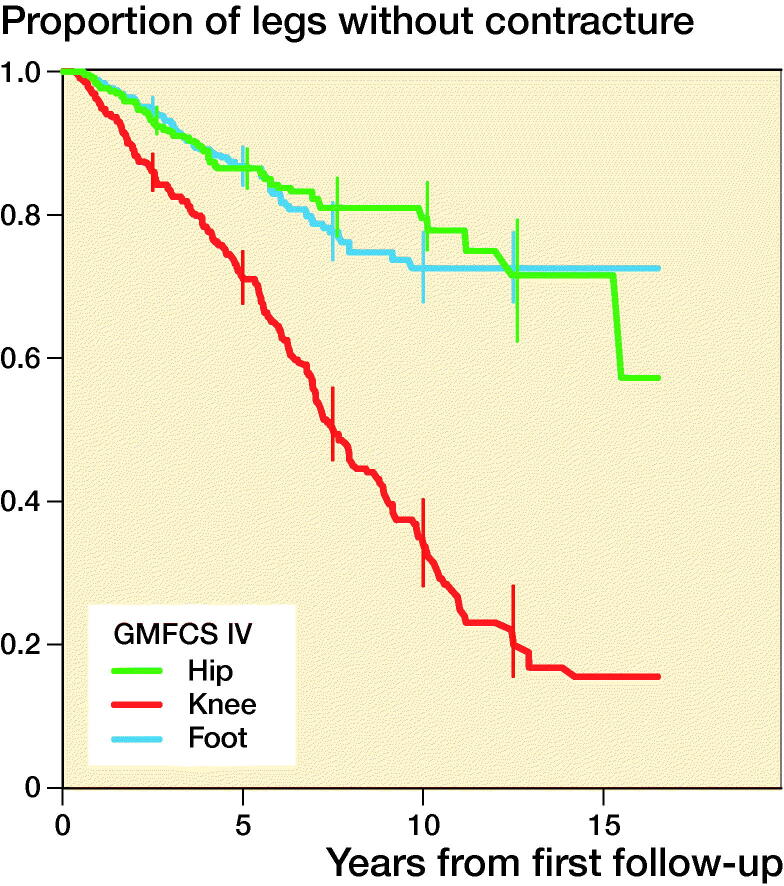

**Figure:**
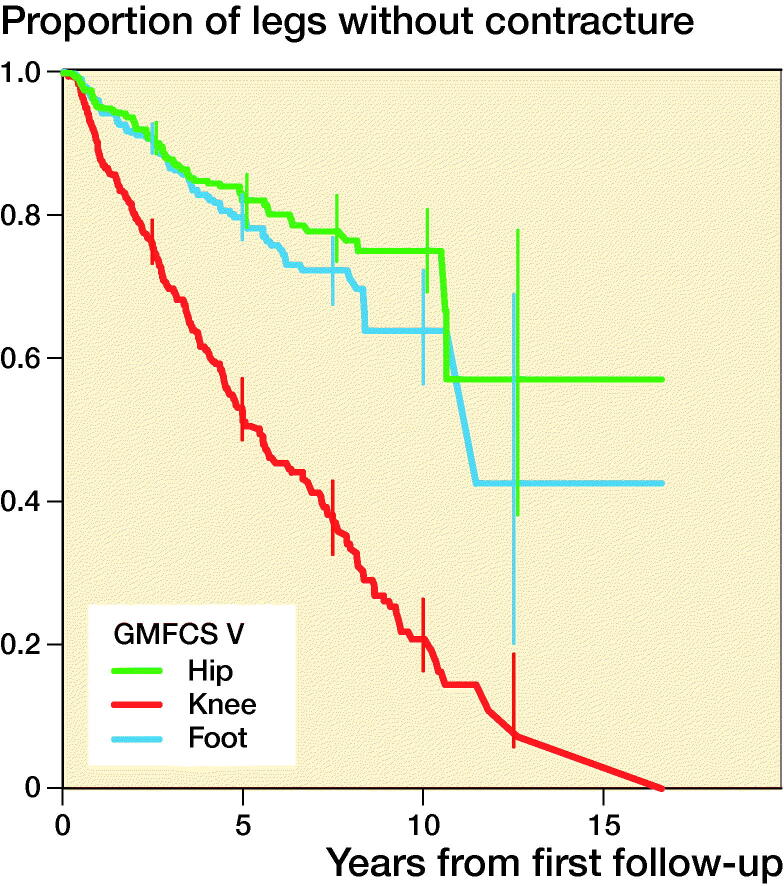

**Table 3.** Proportions of legs free from hip, knee, and foot contracture, stratified by GMFCS level, and 95% pointwise confidence intervals after 10 years of follow-up

GMFCS level	Hip	Knee	Ankle
I	1.0 (0.9–1.0)	1.0 (0.9–1.0)	0.9 (0.9–0.9)
II	0.9 (0.8–0.9)	0.9 (0.9–0.9)	0.8 (0.8–0.9)
III	0.9 (0.8–0.9)	0.5 (0.4–0.6)	0.7 (0.6–0.8)
IV	0.8 (0.8–0.8)	0.3 (0.3–0.4)	0.7 (0.7–0.8)
V	0.8 (0.7–0.8)	0.2 (0.2–0.3)	0.6 (0.6–0.7)

## Discussion

We identified that the first joint contracture to occur in the lower limb involved foot contracture for children with GMFCS level I or II and knee contracture for children with GMFCS level III to V. Children classified with GMFCS level I and II are ambulant whereas those classified with GMFCS level III to V in general rely on wheeled mobility and spend more time sitting with flexed knees. This could explain the sequence in which the contracture presents. This seems to follow the same pattern as previously reported for pain localization in children with CP, in which children with GMFCS level I or II report pain primarily in the feet and those with GMFCS III to V pain in the knees and hips (Alriksson-Schmidt and Hägglund 2016).

These results are consistent with those of a previous study (Nordmark et al. 2009) that reported decreasing ROM from 2 years of age in all lower limb joints. Together, these findings suggest that contracture should be treated early given that ROM seems to decrease over time. Cloodt et al. (2018) found that hamstring length, measured as the unilateral popliteal angle, and dorsiflexion of the foot are strongly associated with the development of knee contracture, whereas spasticity had a significantly smaller effect. Contracture in the lower limbs affects the mechanical position of both the affected and adjacent joints. Preventing normal movements and forces around the joint may increase the risk for additional contractures.

Our study excluded children whose first measurement was at 5 years of age or later. The reason for this was to increase the opportunity of identifying the first contracture and to reflect the natural development of contracture as much as possible because information on treatment and surgery before enrollment in the CPUP was not available in these children. Among the children included, their access to early service and follow-up during their first years of life should be considered. All children with CP in Sweden have access to free health care and interventions from multiprofessional habilitation centers (Alriksson-Schmidt et al. 2017).

Surgery or treatment with SDR and ITB can affect the development of contracture in a specific joint as well as in adjacent joints (McGinley et al. 2012).

There are several strengths and limitations of our study. Classification of subtype was missing in several cases and therefore not included. The contractures were recorded by goniometric measurement, which is a standard measurement in clinical settings but whose reliability varies according to the joint and position (Hancock et al. 2018, Kim et al. 2018). The results included in our study were based on repeated measurements taken by many different examiners, and this may have introduced information bias or measurement errors. In survival analysis, as we used, measurement error can introduce bias. However, this bias has been shown to be small when differences between groups are moderate in terms of hazard ratios (Oh et al. 2018). Furthermore, in some situations in which bias was found to be substantial, bias attenuates observed differences between groups. For our study, this would mean an underestimation of differences in risk of having a first contracture in a specific joint compared with another joint. We validated the data for incorrectly reported measurements to reduce the risk of incorrect outliers. Examiners in the CPUP are encouraged to practice and learn to perform the register’s standardized measurements, which has been shown to be important for reliability (Fosang et al. 2003).

One limitation of our study was the cutoff values for defining contracture. We chose –10° as the cutoff for all joints to be consistent with the reference values used in the surveillance program. Hip or knee extension or foot dorsiflexion of –10° or less causes functional limitations and affects mechanical alignments. To evaluate the effects of this cutoff, we also ran the statistical analyses using less than 0° as the cutoff and found a similar outcome. For children with GMFCS level III to V, the statistical analyses indicated that the conclusion of this study was insensitive to the choice of cutoff values for contracture, that is, 0° or –10°. It is more difficult to draw a conclusion from the trend for GMFCS level I and II because the fewer contractures at these levels of motor function (Nordmark et al. 2009) make it harder to detect statistically significant differences.

Another limitation was the age of the children censored because of surgery. In the CPUP surveillance program, children at risk for hip dislocation have their first soft tissue surgery of the hip early (median age 4 years), which precedes the first foot and knee surgery by 3 and 5 years, respectively. In some cases, hip surgery is likely to occur before confirmed contracture of the hip because of the indication for surgery based solely on lateralization on radiographs (Hägglund et al. 2005). In our study, the operated leg was censored from analyses at the time of the first operation. This may have affected the results, especially for children with GMFCS level IV or V, because many of these children receive their hip operation early and therefore were not included in the analysis.

Botulinum toxin A injection is a common treatment for spasticity in children with CP in Sweden (Franzén et al. 2017) and it could be argued that this interfered with our results. However, given the large number of treated children and the fact that the effect of botulinum toxin is not permanent, these children were included.

The intervals between examinations in the CPUP are based on GMFCS level and age, from twice a year up to the age of 6 years and then every year (GMFCS II–V) or every second year (GMFCS I) (Figure 1). This indicates that some differences between GMFCS levels may stem from differences in detection, probably because of the different number of examinations. Although this may explain some of the differences in the proportion of contractures, it does not explain the fact that contractures occurred in a different sequence, with dorsiflexion first in children with GMFCS level I or II and knee extension first in children with GMFCS level III to V.

Our longitudinal study covers a time period of 24 years and during this period several interventions have changed. Physiotherapy interventions have changed from hands-on sessions to a greater focus on activity and participation. The use of assistive devices and orthoses has increased, and interventions are primarily integrated into the child’s everyday life. There are regional differences in pediatric physiotherapy interventions in Sweden (Degerstedt et al. 2020). More intensive treatment has been associated with better outcome for the children (Storvold et al. 2020). Surgeries are more proactive rather than reactive today and the amount of surgeries has decreased (Hägglund et al. 2005).

Our study indicates that contractures in the lower limb occur early. It seems possible to avoid major surgeries by monitoring patients with CP and treating contractures early on with physiotherapy, orthoses, and botulinum toxin (Hägglund 2005, Novak et al. 2013). Knowledge regarding the occurrence of the first contracture is important to be able to provide early treatment.

An ankle–foot orthosis (AFO) is used widely to facilitate dorsiflexion and to improve walking ability, gait pattern, or foot alignment when standing. In Sweden, 50% of children with CP use an AFO, and the use of AFO starts early and increases with age up to 5 years (Wingstrand et al. 2014). A similar trend has been reported for the development of spasticity of the gastrosoleus muscle, which peaks at age 5–6 years in children with CP (Lindén et al. 2019). Knee orthoses can also be used to increase ROM, but this has not been evaluated widely (Laessker-Alkema and Eek 2016). Most children with CP in Sweden who require a standing device use individually molded hip–knee–ankle–foot orthoses for mechanical alignment of all the segments of the lower extremities.

Botulinum toxin is frequently used in children with CP in Sweden (Franzén et al. 2017). It is most frequently injected into the gastrocnemius muscle to improve gait and increase ROM. The treatment is usually followed by use of orthosis or in some cases serial casting. Botulinum toxin is more frequently used in younger children (Franzén et al. 2017) and its use corresponds to the development of spasticity that increases over the first 5 years of life and then decreases after 6 years of age (Lindén et al. 2019) and GMFCS level I or II. Our results are consistent with this observation that foot contracture occurred first in children with GMFCS levels I–II. Botulinum toxin injection into the hamstring muscles is most common in older children with GMFCS level IV or V (Franzén et al. 2017). Our study showed that knee contracture occurs first in children with GMFCS level IV or V but that contracture occurs at an early age.

It is reasonable to think that a contracture in a joint affects the adjacent joints, which are then at risk for developing contracture. With a knee joint contracture, the hip will not be extended and flexion contracture in connection with abduction or adduction is likely to occur. Knowledge concerning the sequence of the development of contracture in children at different GMFCS levels is crucial for treating the joints early on and preventing the sequence of contractures following the first one.

In conclusion, lower limb contracture occurs first in the foot of children with GMFCS level I and II and in the knee in children with GMFCS level III to V. Early interventions to prevent knee and foot contractures in children with CP should be considered.

### Abbreviations

AFO: ankle–foot orthosis; CP: cerebral palsy; CPUP: Cerebral Palsy Follow-up Program (Sweden); GMFCS: Gross Motor Function Classification System; ITB: intrathecal baclofen pump; KM: Kaplan–Meier; ROM: range of motion; SDR: selective dorsal rhizotomy.

## References

[CIT0001] Agustsson A, Sveinsson T, Rodby-Bousquet E. The effect of asymmetrical limited hip flexion on seating posture, scoliosis and windswept hip distortion. Res Dev Disabil 2017; 71: 18–23. 28987968 10.1016/j.ridd.2017.09.019

[CIT0002] Agustsson A, Sveinsson T, Pope P, Rodby-Bousquet E. Preferred posture in lying and its association with scoliosis and windswept hips in adults with cerebral palsy. Disabil Rehabil 2018: 1–5. 30010440 10.1080/09638288.2018.1492032

[CIT0003] Alriksson-Schmidt A, Hägglund G. Pain in children and adolescents with cerebral palsy: a population-based registry study. Acta Paediatr 2016; 105(6): 665–70. 26880375 10.1111/apa.13368 PMC5071732

[CIT0004] Alriksson-Schmidt A I, Arner M, Westbom L, Krumlinde-Sundholm L, Nordmark E, Rodby-Bousquet E, Hägglund G. A combined surveillance program and quality register improves management of childhood disability. Disabil Rehabil 2017; 39(8): 830–6. 27044661 10.3109/09638288.2016.1161843

[CIT0005] Barrett R S, Lichtwark G A. Gross muscle morphology and structure in spastic cerebral palsy: a systematic review. Dev Med Child Neurol 2010; 52(9): 794–804. 20477832 10.1111/j.1469-8749.2010.03686.x

[CIT0006] Blackman J A, Svensson C I, Marchand S. Pathophysiology of chronic pain in cerebral palsy: implications for pharmacological treatment and research. Dev Med Child Neurol 2018; 60(9): 861–5. 29882358 10.1111/dmcn.13930

[CIT0007] Chan G, Miller F. Assessment and treatment of children with cerebral palsy. Orthop Clin North Am 2014; 45(3): 313–25. 24975760 10.1016/j.ocl.2014.03.003

[CIT0008] Cloodt E, Rosenblad A, Rodby-Bousquet E. Demographic and modifiable factors associated with knee contracture in children with cerebral palsy. Dev Med Child Neurol 2018; 60(4): 391–6. 29318610 10.1111/dmcn.13659

[CIT0009] Degerstedt F, Enberg B, Keisu B I, Björklund M. Inequity in physiotherapeutic interventions for children with cerebral palsy in Sweden: a national registry study. Acta Paediatr 2020; 109(4): 774–82. 31435959 10.1111/apa.14980

[CIT0010] Fosang A L, Galea M P, McCoy A T, Reddihough D S, Story I. Measures of muscle and joint performance in the lower limb of children with cerebral palsy. Dev Med Child Neurol 2003; 45(10): 664–70. 14515937 10.1017/s0012162203001245

[CIT0011] Franzén M, Hägglund G, Alriksson-Schmidt A. Treatment with Botulinum toxin A in a total population of children with cerebral palsy: a retrospective cohort registry study. BMC Musculoskelet Disord 2017; 18(1): 520. 29228927 10.1186/s12891-017-1880-y PMC5725838

[CIT0012] Hägglund G, Andersson S, Duppe H, Lauge-Pedersen H, Nordmark E, Westbom L. Prevention of severe contractures might replace multilevel surgery in cerebral palsy: results of a population-based health care programme and new techniques to reduce spasticity. J Pediatr Orthop B 2005; 14(4): 269–73. 15931031 10.1097/01202412-200507000-00007

[CIT0013] Hancock G E, Hepworth T, Wembridge K. Accuracy and reliability of knee goniometry methods. J Exp Orthop 2018; 5(1): 46. 30341552 10.1186/s40634-018-0161-5 PMC6195503

[CIT0014] Kim D H, An D H, Yoo W G. Validity and reliability of ankle dorsiflexion measures in children with cerebral palsy. J Back Musculoskelet Rehabil 2018; 31(3): 465–8. 10.3233/BMR-170862 28968229

[CIT0015] Laessker-Alkema K, Eek M N. Effect of knee orthoses on hamstring contracture in children with cerebral palsy: multiple single-subject study. Pediatr Phys Ther 2016; 28(3): 347–53. 27027243 10.1097/PEP.0000000000000267

[CIT0016] Lindén O, Hägglund G, Rodby-Bousquet E, Wagner P. The development of spasticity with age in 4,162 children with cerebral palsy: a register-based prospective cohort study. Acta Orthop 2019; 90(3): 286–91. 30907682 10.1080/17453674.2019.1590769 PMC6534199

[CIT0017] McGinley J L, Dobson F, Ganeshalingam R, Shore B J, Rutz E, Graham H K. Single-event multilevel surgery for children with cerebral palsy: a systematic review. Dev Med Child Neurol 2012; 54(2): 117–28. 22111994 10.1111/j.1469-8749.2011.04143.x

[CIT0018] Nordmark E, Hägglund G, Lauge-Pedersen H, Wagner P, Westbom L. Development of lower limb range of motion from early childhood to adolescence in cerebral palsy: a population-based study. BMC Med 2009; 7: 65. 19863779 10.1186/1741-7015-7-65 PMC2774339

[CIT0019] Novak I, McIntyre S, Morgan C, Campbell L, Dark L, Morton N, Stumbles E, Wilson S A, Goldsmith S. A systematic review of interventions for children with cerebral palsy: state of the evidence. Dev Med Child Neurol 2013; 55(10): 885–910. 23962350 10.1111/dmcn.12246

[CIT0020] Oh E J, Shepherd B E, Lumley T, Shaw P A. Considerations for analysis of time-to-event outcomes measured with error: bias and correction with SIMEX. Stat Med 2018; 37(8): 1276–89. 29193180 10.1002/sim.7554 PMC5810403

[CIT0021] Palisano R J, Rosenbaum P, Bartlett D, Livingston M H. Content validity of the expanded and revised Gross Motor Function Classification System. Dev Med Child Neurol 2008; 50(10): 744–50. 18834387 10.1111/j.1469-8749.2008.03089.x

[CIT0022] Pettersson K, Wagner P, Rodby-Bousquet E. Development of a risk score for scoliosis in children with cerebral palsy. Acta Orthop 2020: 91(2): 203–8 31928285 10.1080/17453674.2020.1711621 PMC7144338

[CIT0023] Raja K, Joseph B, Benjamin S, Minocha V, Rana B. Physiological cost index in cerebral palsy: its role in evaluating the efficiency of ambulation. J Pediatr Orthop 2007; 27(2): 130–6. 17314635 10.1097/01.bpb.0000242440.96434.26

[CIT0024] Rosenbaum P, Paneth N, Leviton A, Goldstein M, Bax M, Damiano D, Dan B, Jacobsson B. A report: the definition and classification of cerebral palsy April 2006. Dev Med Child Neurol 2007; 109(Suppl.): 8–14. 17370477

[CIT0025] Smith L R, Chambers H G, Lieber R L. Reduced satellite cell population may lead to contractures in children with cerebral palsy. Dev Med Child Neurol 2013; 55(3): 264–70. 23210987 10.1111/dmcn.12027 PMC4054943

[CIT0026] Storvold G V, Jahnsen R B, Evensen K A I, Bratberg G H. Is more frequent physical therapy associated with increased gross motor improvement in children with cerebral palsy? A national prospective cohort study. Disabil Rehabil 2020; 42(10): 1430–8. 30444146 10.1080/09638288.2018.1528635

[CIT0027] Wingstrand M, Hägglund G, Rodby-Bousquet E. Ankle–foot orthoses in children with cerebral palsy: a cross sectional population based study of 2200 children. BMC Musculoskelet Disord 2014; 15: 327. 25274143 10.1186/1471-2474-15-327 PMC4192348

